# Characterization and Potential Application of Bromelain from Pineapple (*Ananas comosus*) Waste (Peel) in Recovery of Silver from X-Ray Films

**DOI:** 10.1155/2021/9964337

**Published:** 2021-11-17

**Authors:** Muhammed Seid Anbesaw

**Affiliations:** Wollo University, School of Bio-Science and Technology, Department of Biotechnology, Dessie, Ethiopia

## Abstract

Bromelain is a proteolytic enzyme, which is predominately found in all parts of a pineapple plant (*Ananas comosus*). It has immense application in the pharmaceutical industry as well as in food, cosmetic, and leather industries. However, bromelain from pineapple fruit peels is a less explored source for making valuable products. Therefore, the objective of this study was to characterize and investigate the potential application of bromelain enzyme extracted from pineapple juice processing waste peels in gelatin hydrolysis and removal of silver from X-ray films. Extraction of bromelain was performed with a 1 : 2 ratio (w/v) of the extraction mix, pineapple fruit peel, in phosphate buffer (pH = 7.0). The activity of a crude enzyme was 7.2 U/ml, and it was active in a broad range of pH (2.5–12) and temperature (25–85°C) without losing its activity. This implies that the enzyme is heat tolerant. The optimum temperature and pH of the enzyme were recorded at 70°C and pH 7.0, respectively. At optimum conditions (70°C and pH 7.0), complete hydrolysis of the gelatin layer from X-ray film was observed at 30 and 34 seconds, respectively. The enzyme was repeatedly used more than 50 times without significant loss of its activity. Using a minimum concentration of bromelain (3 ml = 21.6 U) along with phosphate buffer (37 ml), it is possible completely to remove gelatin within 210 seconds. The properties of the enzyme showed that it has promising potential industrial applications for repeated utilization of the enzyme in both silver recovery and recycling of the X-ray film base.

## 1. Introduction

Silver is one of the precious and novel metals. It is used in large amounts for various purposes, especially in the photographic industry [1]. Photographic and X-ray films contain silver particles spread on the surface linked to the gelatin layer [2]. Due to this, photographic and mainly X-ray films have been taken as a good source of silver recovery. The amount of silver on X-ray film varies from 1.5 to 2.0% by weight [1, 3]. It is reported that 25% of the world's silver needs are supplied by recycling, of which 75% is obtained from photographic waste [1]. Due to the increased demand for silver in the world, recent attention is focused on X-ray/photographic films as one of the secondary sources of silver as they contain a considerable amount of silver in them.

Various methods of silver recovery from X-ray/photographic films have been utilized. Of these, burning of the films directly, oxidation of metallic silver following electrolysis, stripping the gelatin layer using different chemical solutions, and enzyme hydrolysis of gelatin are worth mentioning [1, 3–6]. The most common process to recover silver from the waste/used X-ray film is to burn the film in a furnace and retrieve the precious metal from the ashes. The traditional method of silver recovery, by burning the films directly, generates an undesirable foul smell, causes environmental pollution, and destroys the polyester film base and the gelatin coat on the film. However, this method is “rather expensive because of the cost of maintaining the furnace and treating the soot and foul-smelling smoke” [7]. Similarly, stripping the gelatin-silver layer by chemical methods using various chemicals and reagents causes environmental hazards. Moreover, these methods are time-consuming and expensive and pose an industrial safety problem. Therefore, the methods applied to recover silver from X-ray/photographic waste should be cost-effective and sound to the environment.

Recovery of silver from X-ray/photographic waste using enzyme technology can be an alternative. Since the thin-coating (emulsion) layer on X-ray film contains silver and gelatin, it is possible to hydrolyze the gelatin layer using proteolytic enzymes and remove the silver [1, 3]. The enzymatic hydrolysis of the gelatin layers on X-ray offers twofold advantages that enable both the recovery of silver and recycle of the polyester base. As a result, in recent years, enzymatic methods using microbial proteases are being explored as alternatives to the burning and oxidation methods of silver recovery from photographic/X-ray films [3, 7–12]. Enzymatic processes are more specific and remove the gelatin layer from X-ray film in a few minutes without damaging the polyester film base.

Even though microbes are the best alternative source of enzymes used for different applications, maintaining the microbe at its optimum condition is difficult. Their growth medium cost is also one of the factors for the production of the enzyme. Therefore, there is a need for an alternative enzyme sources and low-cost materials to produce enzymes including agro-industrial residues from food industries. Pineapple is used for different purposes like juice production and about 30–42% of the pineapple mass becomes a byproduct and generates as a waste in the form of crown, bottom, leaf, core, peel, etc. [13, 14]. These by-products have a low commercial value; they are directly disposed and cause environmental pollution. However, these wastes are a potential source for bromelain enzymes that can be used for different applications.

In the past few years, many attempts have been made to study the biochemical properties and enormous potential applications of bromelain in different sectors. The enzyme has been used widely in food [15], cosmetic [16], textile [17], leather [15], medical and pharmaceutical [18], and other industries. However, no one presents the importance of bromelain in the recovery of silver from waste X-rays and photographic films except Radha and Arun [2]. Their work has been also focused only on the extraction of bromelain from pineapple apex, but not from other parts of the plant. Furthermore, no reports are available in the scientific literature related to silver recovery from X-ray films by using pineapple juice processing waste (peels). Hence, the objective of this study was designed as a novel, simple, fast, cheap, and pollution-free method of recovering silver from X-ray films using bromelain extracted from pineapple juice processing waste (peels).

## 2. Materials and Methods

Waste X-ray films were collected from different health centers of Dessie town including Dessie referral and private hospitals and other clinics. The pineapple juice processing waste (peel) used for the extraction of bromelain was collected from local juice houses into clean plastic bags. The sample was brought to the laboratory and kept in the refrigerator at 4°C until further use. All the experiments conducted in this study were done in the Biotechnology Laboratory at Wollo University.

### 2.1. Extraction of Bromelain from Pineapple Peel

Since phosphate buffer (pH 7.0) maintains the system pH close to the optimum pH of bromelain and keeps normal enzyme activity [19], this buffer is also used to extract crude bromelain from pineapple juice processing waste (peel) used for this entire work. About 500 gm of waste pineapple peel was added in 1 L distilled water (1 : 2 w/v), and the mixture was blended using Moulinex LM241027 juice maker/extractor, France, with 14200 rpm for 5 minutes at room temperature. Then, as described by Ketnawa et al. [14], the homogenized solution was filtered using two layers of muslin cloth followed by centrifugation at 10,000 g for 20 min at 4°C to remove insoluble material using a tabletop high-speed centrifuge HC-20C model. Finally, the clear supernatant was transferred into a clean bottle and kept at 4°C which was used as a crude bromelain enzyme and used for further tasks.

### 2.2. Enzyme Assay

Proteolytic enzyme activity of bromelain was determined using gelatin as a substrate with a slight modification of the method described by Nakiboglu et al. [3] and Shankar et al. [1]. Briefly, an aliquot of 500 *μ*l of the crude enzyme was mixed with 500 *μ*l of phosphate buffer (0.1 M, pH 7.0) containing 1% (w/v) gelatin and incubated for 30 min at 70°C in a digital water bath (XMTD-204 model). The reaction mixture was 500 *μ*l of 1% gelatin dissolved in 0.1 M phosphate buffer (Na_2_HPO_4_/NaH_2_PO_4_), pH 7.0, and 500 *μ*l of crude enzyme extract. Since enzyme reactions are required at accurately controlled temperatures, the reaction mixture was prepared in Eppendorf tubes and incubated in the water bath at 70°C for 30 min. After 30 minutes of incubation, 500 *μ*l of 10% trichloroacetic acid (TCA) was added to stop the reaction. Enzyme blank (control) was processed by adding the same amount of enzyme after TCA was immediately added, and the tubes were allowed to stand for 20 min at room temperature to facilitate the unreacted gelatin precipitation. The precipitated protein was removed by centrifugation at 10,000 rpm for 5 min. From the clear supernatant, 500 *μ*l was transferred to a clean test tube. Then, 2.5 ml of 0.5 M Na_2_CO_3_ and 500 *μ*l 2 N Folin–Ciocalteu's phenol reagent (1 : 10 diluted) were added to the solution and the solution was immediately mixed with vortex and kept at room temperature for 20 minutes. Finally, absorbance reading was measured using Ultraviolet Visible Spectrophotometer (UV-7804C model, China) at 660 nm. All experiments were performed in triplicate. The enzyme activity was expressed in units (U) and calculated by using the tyrosine standard calibration curve. One unit of bromelain enzyme was defined as the amount of enzyme that releases 1 *μ*g of tyrosine per ml of crude extract per minute under standard conditions at 70°C.

### 2.3. Preparation of Tyrosine Standard Curve

Zero-point five molars (0.5 M) of Na_2_CO_3_, 2 N Folin reagent (1 : 10 diluted), distilled water, and 1 mg/ml of Tyrosine stock solution were used for the preparation of the curve. According to the intended working concentration of tyrosine, a required amount of stock solution was added in each test tube except the blank, and distilled water was added in each tube to make the volume 1000 *μ*l including blank. Then, 2.5 ml of 0.5 M Na_2_CO_3_ was added in each test tube including blank, and the mixtures were kept at room temperature for 10 min. After 500 *μ*l of 2 N Folin reagent was added in each test tube including blank, the solution was mixed immediately and kept for 30 min at room temperature. Finally, the optical density (OD) was measured at 660 nm using a UV-7804C spectrophotometer. Based on the above procedures and experimental results (data not shown), the standard curve was plotted. To determine the crude bromelain activity, calibration curve equation (*y* = 0.0102*X* + 0.0206) was generated from the standard curve with the regression coefficient of *R*^2^ = 0.9977.

### 2.4. The Effect of Temperature on Enzyme Activity

The effect of temperature on enzyme activity was investigated by incubating the enzyme in an adjustable digital water bath from 25 to 90°C at intervals of 5.0 units. The activity of the enzyme was evaluated based on the time required for complete removal of gelatin from X-ray film. Since the reaction mixture (enzyme) cannot maintain the required temperature (adjusted temperature bath) after immediately incubating in the bath, the enzyme was incubated about 5–7 minutes in the bath before the film was added. The activity of the temperature at which the highest activity was observed was taken as optimal (100%). Thermostability of the crude enzyme was examined by incubating it at 70°C for 30, 60, 90, and 180 minutes followed by determination of residual activity at each incubation time. The nonheated crude enzyme was taken as 100%.

### 2.5. The Effect of pH on Enzyme Activity

The effect of pH on enzyme activity was determined by assaying its activity at different pH values within the range of 2.5–12.5 with an increment of 0.5 pH units in the presence of 0.1 M concentration of the following buffers: glycine/HCl (pH 2.5), sodium citrate (pH 3–6), phosphate (pH 6.5–7.5), Tris/HCl (pH 8 and 8.5), and glycine/NaOH (pH 9–12.5). All pH values were adjusted using a pH meter (Sx725 model). Experimental activity was carried out by mixing an equal amount of crude enzyme and the desired buffer (10 ml : 10 ml). The enzyme activity was measured following the procedure described (Section 2.6) at constant temperature (70°C) and the activity of the pH at which the highest activity was taken as optimal (100%). pH stability was determined by preincubating the crude enzyme in buffer having pH values of 3–11 for 3, 6, 12, 24, and 48 hr. The residual activity of the enzyme was calculated by the following formula [20]: residual activity = activity of the enzyme after incubation/activity of the enzyme before incubation) × 100. Preincubation of the reaction mixtures was carried out by mixing an equal amount of crude enzyme and the desired buffer.

### 2.6. Removal of Gelatin and Silver from Used X-Ray Film

Used X-ray films were washed with tap water to remove the dust from the dust and sun-dried. Then, the films were cut into 4 cm × 4 cm pieces, each weighing 0.397 gm. Four beakers, each with 400 ml volume, were labeled as test beakers 1, 2, and 3, and control beakers were prepared. Test beakers containing crude bromelain (20 ml = 144 U) and the control beaker containing only phosphate buffer (20 ml) were dipped 5–7 cm depth in the preadjusted digital water bath (70°C) until the desired temperature was maintained. Then, the films were immediately immersed in all beakers including the control beaker. The reaction was proceeded with continuous hand (manual) shaking until the gelatin and silver were completely removed from the film. Complete removal of the gelatin layer from the X-ray film was monitored visually until the clean transparent film resulted, which was performed by picking the films with forceps. The time taken for the complete removal of the gelatin layer from the X-film was recorded [10].

### 2.7. Reusability of the Enzyme for Gelatin Removal from X-Ray Film

Reusability (how many times could the enzyme be reused) of the enzyme towards the removal of gelatin and silver from X-ray films was evaluated using 7.2 U/ml of the enzyme at 70°C and pH 7.0. After the clean and transparent X-ray film was observed, enzyme-treated X-ray film was picked out (removed) from the reaction mixture. Then, the new film (the untreated one) was added to the same enzyme solution and incubation was continued until complete removal of gelatin was observed. The process was repeated until the enzyme became inactive (loss of its activity) towards gelatin hydrolysis. The time required for complete gelatin removal in each case was noted [10].

### 2.8. Effect of Enzyme Concentration and Time Course of Gelatin Hydrolysis

Effect of enzyme concentration on gelatin hydrolysis was determined with enzyme concentrations ranging from 0.00 ml (0.00 U) to 100 ml (720 U). Phosphate buffer at pH 7.0 was used to dilute the enzyme and the experiment was carried out at the optimum temperature of the enzyme, 70°C. The time course of gelatin layer removal from X-ray film was monitored visually, and the specific time of gelatin removal was recorded accordingly.

### 2.9. Evaluation of the Active Period (Activity, Shelf Life Time) of the Enzyme

The active period of the crude enzyme was evaluated by withdrawing the sample from storage (4°C) at 2, 4, 8, 16, and 30 days. The crude enzymes taken from each day were evaluated for their gelatin removal efficiency at the optimum temperature (70°C) and pH (pH 7.0) of the enzyme. Gelatin and silver removal efficiency of the enzyme was evaluated by recording the exact time required for complete removal of gelatin and silver from the film.

### 2.10. Weight Loss of X-Ray Films during Removal of Gelatin and Silver

Rough estimation of the silver content that can be recovered from X-ray films was calculated by considering the weight loss due to the hydrolysis of gelatin and removal of silver from the used X-ray film. A 4 × 4 cm^2^ cut or 16 cm^2^ of the film was weighed before and after hydrolysis of the gelatin layer using an analytical sensitive balance. The weight difference was calculated and expressed in percentage. This value was considered as the solid particles (silver) removed from the film, which were previously coated on the film. Since the sizes of X-ray films are varied according to the manufacturers, it is difficult to estimate the amount of silver per X-ray films. Therefore, the amount of silver content that can be recovered per 1 cm^2^ film was estimated as described by [7].

## 3. Results and Discussion

### 3.1. Extraction of Bromelain Enzyme

**Figure 1:**
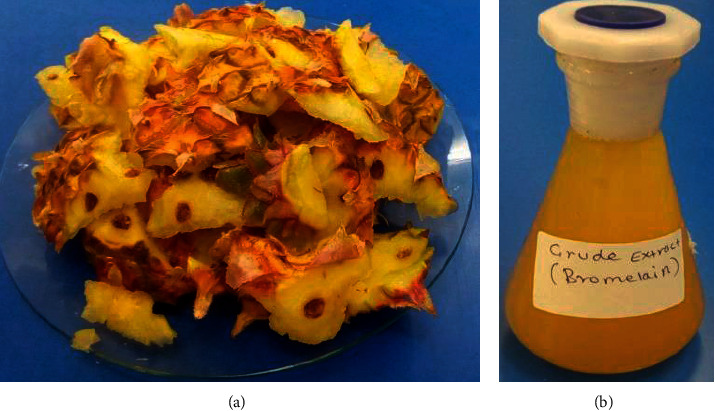
(a) Pineapple juice processing waste (peels). (b) Crude extract of bromelain enzyme.

As described earlier (Section 2.1), extraction of bromelain was performed with 1 : 2 ratio of extraction mix (i.e., pineapple fruit peel: phosphate buffer, pH 7.0). Based on this extraction mix, 275 ml (1980 U) of the crude enzyme (Figure 1(b)) was obtained from 100 gm of pineapple fruit peel (Figure 1(a)). This implies that 137.5% of an enzyme can be recovered from 100 gm of the peel. This extra percentage of enzyme recovery indicates the presence of a naturally existing liquid component of the peel. Of the total biomass of the peel, 17.5 gm of wet solid biomass was recovered as residue. This residue contains a high amount of fiber, and it might be useful for different purposes including as a substrate for the cultivation of industrially important fungi in solid-state fermentation. Since agricultural wastes are rich sources of carbon, nitrogen, and minerals [21], the residue (fiber) can be used for paper and paperboard production as well as used as a media supplement for the cultivation of microorganisms. Ketnawa et al. [14] also reported that 163.5 ml and 154.5 ml crude extract (bromelain) were obtained from 100 gm of pineapple peels extracted with distilled water from two cultivars, namely, Nang Lae and Phu Lae, respectively. In another study of Ketnawa et al. [22], 152 to 162 mL bromelain extract was obtained from 100 gm of the peel of Phu Lae extracted with phosphate buffer. The result implies that extraction yield variation was observed (17 to 26 ml in the first case and 14.5 to 24.5 ml in the second case). The observed variation of this little volume of extract might come from the filtration efficiency, the type of extractant (buffer) used, or liquid content variation of the pineapple plant cultivars used in the present study and the above two studies. This speculation was also supported by the study conducted by Ali et al. [23]. The results showed that the chemical composition variability and fiber content of the pineapple plant also varied based on geographical origin, cultivar variety, and climatological conditions.

Under the standard bromelain enzyme assay conditions described earlier (Section 2.2), the activity of crude bromelain enzyme extracted from pineapple fruit peel was 7.2 U/ml (calculation not shown). Mohan et al. [24] also reported that the activity of crude bromelain extracted by distilled water from pineapple flesh and peel was found to be 4.71 U/ml and 4.52 U/ml, respectively. Ketnawa et al. [22] also investigated that the activity of crude bromelain extracted by phosphate buffer from two pineapple cultivar fruit peels (Nang Lae and Phu Lae) was recorded as 2.76 U/ml and 5.97 U/ml, respectively. Furthermore, 0.002 U/mL to 0.0085 U/mL of bromelain activity was reported when the pulp and stem part of pineapple was extracted with distilled water, respectively [25]. All above studies revealed that the activity of bromelain can be varied if the extractant buffer and the parts of pineapple used for bromelain extraction varied.

The pH value of the crude extract (bromelain) studied under this study was found to be around 3.98 and 5.18 units extracted with distilled water and phosphate buffer (pH 7.0), respectively. Similarly, Chaurasiya and Hebbar [19] also reported that crude bromelain extracted with similar buffer used in this study (0.1 M phosphate buffer) characterized by a pH value around 5.0. Furthermore, Ketnawa et al. [22] investigated that crude bromelain extracted from pineapple peel obtained from two cultivars using phosphate buffer characterized by a pH value of 5.15–5.83. This implies that the crude extract is mainly bromelain obtained from pineapple fruit and its byproduct is acidic. This is probably because of the presence of organic acids in the extract and causes the pH value to be lower. The work of Bartolome et al. [26] and Ketnawa et al. [14] also proved that the existence of citric and maleic acid in the pineapple plant extract. This makes the pH value of the extract to be lower and found in the range of 3.89–4.8 if the enzyme is extracted with distilled water [14], deionized water [26], or without the addition of any extractant [27]. All the above results and other related investigations indicate that crude bromelain is acidic in nature and its pH value may be varied based on the nature of the extractant, mixed ratio (extractant and pineapple material), the strength of the buffer, and the part of pineapple used as a source of bromelain.

### 3.2. Gelatin Removal Efficiency of the Enzyme

Despite human health and environmental pollution problems, numerous works have been conducted to remove gelatin from X-ray films by using chemical methods [5, 28]. Similarly, considerable studies have been also investigated to remove gelatin using enzymes from microorganisms. However, the production cost of the enzyme and maintaining of culture condition for the cultivation of the organism is not cost-effective and the gelatin removal efficiency of the enzymes is also extremely slow and nonefficient when compared to the enzyme used in the present study. The enzyme studied under this study, which was extracted from pineapple juice processing waste peels, can remove gelatin completely within 20 and 34 seconds under optimum conditions (pH 7.0 and 70°C) with 40 ml (288 U) and 10 ml (72 U) enzymes, respectively.

There are numerous studies (Table 1) that also reported that gelatin can be removed from X-ray films using enzymes obtained from different microorganisms. The results showed that the enzyme used in this study (crude bromelain) which was extracted from pineapple juice processing waste (peel) is extremely efficient to remove gelatin from X-ray film and recover silver when compared to other protease enzymes reported before (Table 1). Based on this study and the previous other investigations (Table 1), it is possible to prove that enzyme from plant source especially from pineapple is more effective than enzymes obtained from microorganisms for gelatin hydrolysis purpose. Besides this, all investigations proved that enzymatic hydrolysis of gelatin from X-ray films enables not only the recovery of silver but also the X-ray films, which can be recycled without damaging it.

Conclusively, to date, no microbial and plant enzymes have been reported that can remove gelatin from X-ray films within the reported time interval or incubation period of the present study (20 to 34 seconds). This includes bromelain enzymes extracted from pineapple cultivars found abroad in Ethiopia. A representative study conducted by Radha and Arun [2] indicates that crude bromelain extracted from the waste apex of pineapple needs 35 minutes to remove gelatin from X-ray films. This implies that crude bromelain extracted from pineapple fruit peel was significantly effective in gelatin hydrolysis. This can be explained by the fact that the protein content in the fruit peel is found to be more, which is directly correlated to the amount of bromelain present, meaning that the amount of protease is more in the fruit peel extract. Arumugam et al. [34] also reported a similar argument in this regard. Therefore, the observed great difference in gelatin removal efficiency of bromelain extracted from pineapple cultivars found abroad and found in Ethiopia might be the accumulation of bioactive compound concentration difference in pineapple cultivars, and the agro-ecological difference may also probably make this difference. This prediction is also argued by Ali et al. [23]. Their reports showed that geographical origin, cultivar variety, and climatological conditions make pineapple plants vary in chemical composition and content.

**Figure 2:**
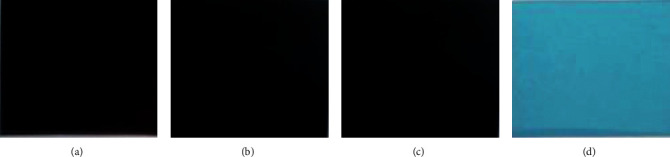
Removal of gelatin and recovery of silver from X-ray films from crude bromelain. Used X-ray films (a) and (b) were collected from Dessie Referral Hospital and (c) and (d) were collected from other health centers that have not used digital X-ray processing machines. Used X-ray films, (a) and (c), were control treated with phosphate buffer only, whereas (b) and (d) were enzyme (20 ml = 144 U) treated films.

As presented in Figures 2(a) and 2(b), waste X-ray films collected from health centers, which are used in the digital X-ray processing machine (Dessie Referral Hospital) treated with bromelain, could not be washed out or no hydrolysis of gelatin was observed until 48 h incubation. This implies that X-ray films processed in digital X-ray machines are different from analog (manual X-ray processing) machines and image processing might be also involving enzyme inhibitor chemicals. The result clearly shows (Figure 2(d)) that X-ray films processed in a nonautomated process (manual) can be washed with bromelain easily.

### 3.3. The Effect of Temperature on Bromelain Activity

**Figure 3:**
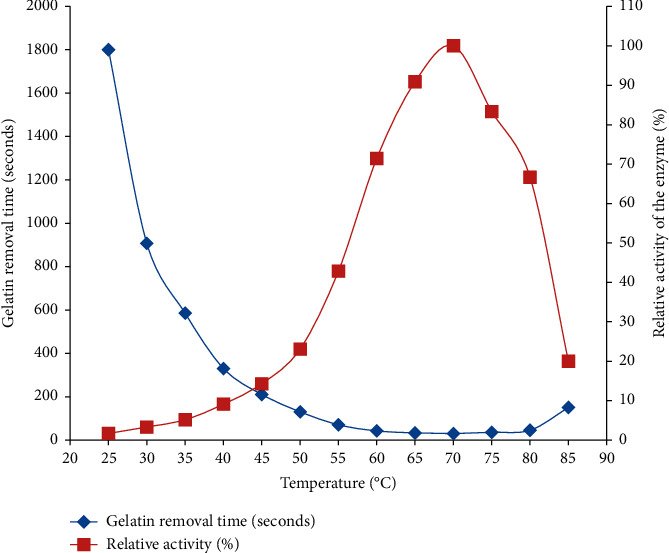
The effect of temperature on bromelain activity on removal of gelatin from X-ray film (reaction mix 20 ml., i.e., 20 ml enzyme or 144 U) at a constant pH value of 7.0.

The enzymes to be used in industrial applications must be active at a wide range of temperatures. With this scenario, the enzyme used in this study can be a potential candidate for an industrial process that needs the enzyme having the ability to hydrolyze proteins, especially gelatin, within 25–80°C temperature range. As presented in Figure 3, complete gelatin hydrolysis was increased with an increase in temperature and reached a maximum at 70°C. Therefore, this temperature is considered as the optimum temperature of crude bromelain extracted from waste pineapple peels.

Complete removal of gelatin from X-ray films was noticed even at lower (25°C) and high temperature (90°C) values. Within the temperature range of 55–80°C, the enzyme retained more than 40% of its maximum activity (Figure 3). Thus, the enzyme used in this study was more thermotolerant and can be used in the industrial process, which is performed under relatively high temperature, because the enzyme was stable enough to be used at 70°C. This reflects that the enzyme possesses remarkable heat-stable properties, in which most enzymes are destroyed or denatured. However, below 50°C, the enzyme retained <20% of its activity. The observed active hydrolysis of gelatin at a lower temperature (25°C) provides another interesting finding to use this enzyme at room temperature without the requirement of heat energy to remove gelatin from the film. At this lower temperature (25°C), removal of gelatin can be accomplished within 30 minutes, which is comparable to other enzymes that can be used for gelatin hydrolysis at a higher temperature (50–60°C) requiring an energy-intensive process [30, 33].

At higher temperatures (>90°C), the enzyme decreased its activity and became inactive due to the denaturation of its structure. In this study, the enhanced enzyme activity at the upper temperature is a unique finding. Similarly, Ketnawa et al. [14] also proved that crude bromelain becomes inactive at this temperature value. Enhanced enzyme activity at the upper temperature is a unique finding from this study. Other related investigations [35, 36] showed that bromelain works optimally at 55 and 63°C, respectively. Similarly, Ketnawa et al. [14] and Koh et al. [17] also reported that 60°C was the optimum temperature for bromelain activity.

**Figure 4:**
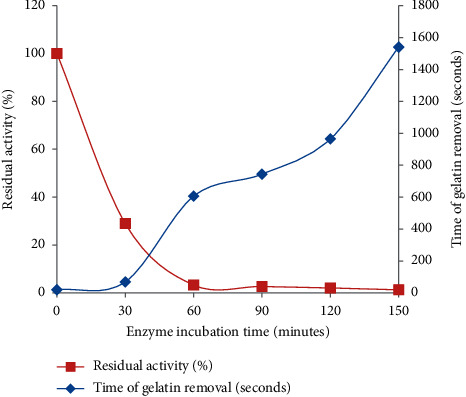
Stability of the enzyme at 70°C (reaction mix 40 ml or 288 U).

Thermal stability of the enzyme indicated that only 28.96% of the enzyme residual activity was retained after 30 minutes of preincubation. The residual activity of the enzyme after 1 hr preincubation inferred that the enzyme was not stable and dramatically decreased its activity. The result revealed that the enzyme could not be stable at its optimum temperature for a long period (>1 hr) of incubation (Figure 4). A related study [37] shows that stem bromelain can retain 18% of the original activity after 3 hr of incubation at 60°C, pH 7.0.

### 3.4. The Effect of pH on Bromelain Activity

**Figure 5:**
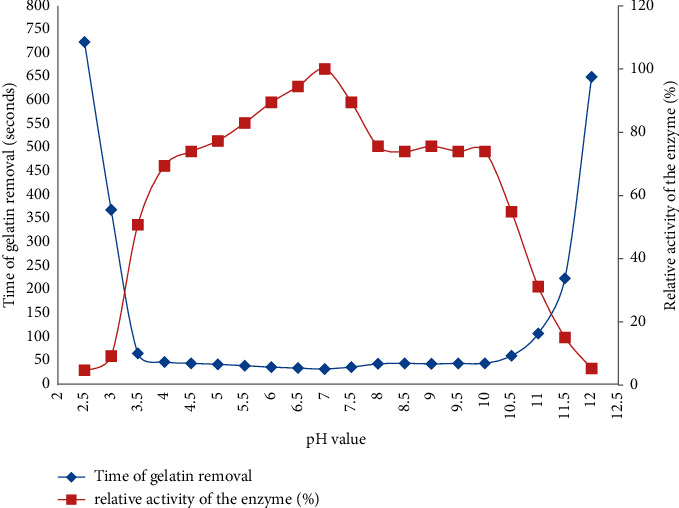
The effect of pH on enzyme activity (reaction mix 20 ml, i.e., 10 ml buffer + 10 ml enzyme or 72 U) at constant temperature of 70°C.

Complete gelatin removal was observed over a wide pH range (2.5 to 12.0) with maximum gelatin hydrolysis at pH 7.0 (Figure 5). The activity of bromelain was found to increase proportionally with the increase in pH from 2.5 to 10.5 with a drop in activity beyond pH 12. The result shows that the time required to complete gelatin hydrolysis from X-ray film at the optimum pH (7.0) was 34 seconds. The gelatin removal efficiency of bromelains at a wide range of pH ranges was probably due to the presence of a complex mixture of proteolytic enzymes in bromelains with their unique properties to work on at different pH values. Under very acidic (<pH 2.5) and alkaline (>pH 12.0) conditions, partial hydrolysis of gelatin was observed. Relatively, the enzyme was highly active and shows >50% of its activity within 3.5 to 10.5 pH range. A similar result also investigated that the highest activity of bromelain was recorded at pH 7.0 [14, 17, 19, 38, 39]. Similar studies also indicated that fruit bromelain has an optimum pH at 7.5 [40] and around 6.8 [41]. The above results indicated that neutral pH 7.0 is a conducive environment for crude bromelain to be active.

Relative activity: activity measured relative to that of the time of gelatin removal obtained at pH 7.0 and 70°C, which is considered as 100%.

As shown in (Table 2), the enzyme shows its remarkable pH stability and retained its residual activity after 3 h preincubation for gelatin hydrolysis test. In all pH values, the enzyme was retained >75.5% of its residual activity after 3 h preincubation. The enzyme was retained >50% of its original activity in all pH values (3–11) after a 12 h preincubation test for gelatin hydrolysis. In contrary to the optimum pH (7.0) that retained 87.18% of residual activity, the enzyme possessed maximum (92%) residual activity at pH 10 after 48 h preincubation for the gelatin hydrolysis test. The enzyme was retained >20% of its residual activity after 48 h preincubation in all pH values except at pH 8. At this pH value, the enzyme retained only 9.38% of its residual activity. This can be explained by the fact that tris(hydroxymethyl)aminomethane acts as a competitive inhibitor of some enzymes, and it chelates calcium and other essential metals. That is why Tris buffer is not suitable and used to remove gelatin from waste X-ray film by bromelain after a long period. This might be bromelain is a calcium-dependent enzyme.

### 3.5. The Effect of Bromelain Concentration on Gelatin Removal Efficiency

**Figure 6:**
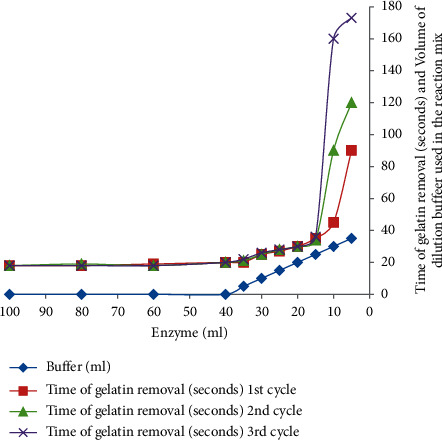
Effect of enzyme concentration on the time course of gelatin hydrolysis at 70°C and pH 7.0.

The effect of bromelain concentration on the hydrolysis of gelatin was studied and the rate of gelatin hydrolysis increased with the increase in bromelain concentration, which means that at higher enzyme concentration, a greater gelatin hydrolysis rate was observed. Effect of time on gelatin removal shows that at 20 seconds essentially all gelatin and silver grains on X-ray film were completely stripped out from X-ray films using 40 ml (288 U) of the enzyme. As can be seen in Figure 6, when the bromelain concentrations ranged between 40 ml (288 U) and 100 ml (720 U), the enzyme shows a linear pattern of gelatin hydrolysis because complete removal of gelatin was observed by two seconds difference (18 to 20) within these enzyme concentration ranges. As the time of enzyme concentration decreased, the enzyme took a longer reaction time for complete gelatin removal. Complete removal of gelatin from X-ray films could not be observed when the concentration of bromelain <3 ml (0.54 U) in the reaction mix (40 ml). Ingale et al. [8] reported that gelatin can be removed completely within 12 minutes using 10 U of the enzyme. Singh et al. [42] also reported that gelatin can be removed within 24 minutes using a similar enzyme concentration (10 U). Furthermore, complete removal of the gelatin layer was observed after 2 minutes using 1000 U [4] and after 33 minutes using 6.9 U of the enzyme [11]. Therefore, the results showed that the enzyme used in this study is extremely efficient to remove gelatin from X-ray films within a short period as well as with a low concentration of enzyme.

### 3.6. Stability and Reusability of the Enzyme

**Figure 7:**
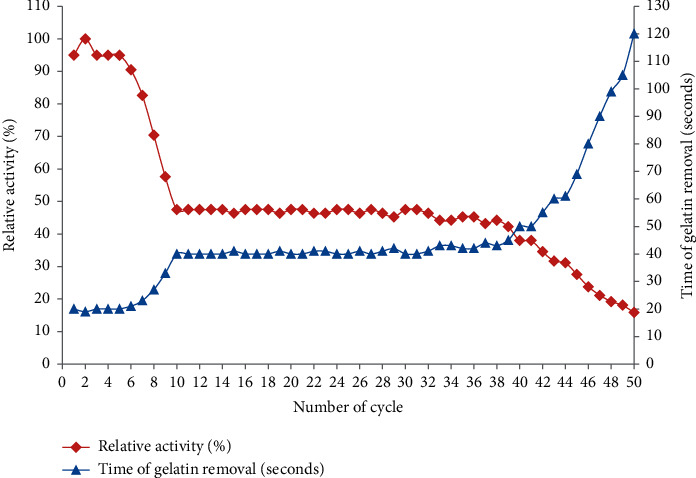
Reusability of crude bromelain for gelatin hydrolysis from waste X-ray films.

The enzyme (288 U) can be reused efficiently 50 times by retaining its normal activity without any additive of protective agents (glycerol and propylene glycol) against thermal inactivation. However, gradually the enzyme lost its activity after the 50th cycle reuse. The minimum time required to remove gelatin from X-ray films was 19 seconds (2nd cycle), whereas 120 seconds (50th cycle) was the maximum. The total time recorded to accomplish 50 times reused was <38 minutes. Gelatin hydrolysis time of bromelain remained constant from the 10th to 39th cycles and 40 to 45 seconds required to complete hydrolysis. The enzyme decreased its relative activity from the 1st to 9th cycles (100–57.6%) and after the 40th to 50th cycle (50–15.8%). In between these cycles (10th to 39th), its relative activity became stable (47.5–42.2%) (Figure 7). Similar works have been done [1, 7] to remove gelatin from X-ray films, but the enzymes can be reused four times. Cavello et al. [11] also reported that proteases from *Purpureocillium lilacinum* LPS no. 876 can be reused three times to remove gelatin from waste X-ray films. Until to date, no reports are available in the literature that the enzyme from any source can be reused 50 times to remove gelatin from X-ray films. Since the enzyme used in this study shows good reusability and prolonged stability, it can provide an economic gain and is also used constantly in both batch and continuous reactors, especially in the gelatin hydrolysis process.

### 3.7. Storage Stability (Active Period) of the Enzyme

**Figure 8:**
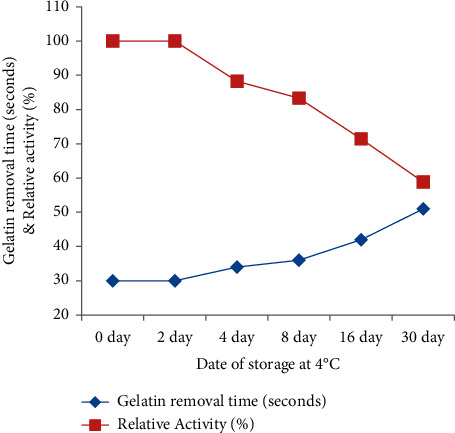
Active period of the enzyme after extraction was performed and kept at 4°C.

The activity shelf life of the enzyme on its gelatin and silver removal efficiency was determined by withdrawing it from its storage (4°C). As presented in (Figure 8), the enzyme was active to remove gelatin and silver from X-ray film even after 30 days of its extraction period. The enzyme shows 100% residual activity after extraction was performed at 48 h. Declining enzyme activity was observed when the enzyme was stored for a longer period. At 30 days of storage, 58.8% residual activity was recorded. This implies that the enzyme has the ability to hydrolyze gelatin even if it is stored for more than a month. Furthermore, the enzyme could retain more residual activity if it is purified further. As Bhattacharya and Bhattacharyya [43] reported, crude bromelain activity could retain 50% of its activity after 2 months when it was kept at 4°C without any preservatives. All results indicate that to achieve higher activity of bromelain, the extract should be used as soon as possible or better to use fresh extract. This truth was also reported by [19].

### 3.8. Weight Loss and Recovery of Silver

Weight loss due to the hydrolysis of gelatin and removal of silver from X-ray film was calculated and expressed in percentage. A 4 × 4 cm^2^ cut or 16 cm^2^ of used X-ray film was weighed before (0.397 gm) and after hydrolysis of the gelatin layer (0.392 gm). The weight difference (0.397 gm–0.392 gm) of the film shows that 0.005 gm or 1.259% of its weight. Hence, the film has lost 1.259% (0.005 gm) of its original weight. Based on this data, if the 1 kg film is washed out, 12.59 gm silver could be recovered. Parpalliwar et al. [7] reported that 0.49 mg/cm^2^ silver content can be recovered from the used photographic films. The result obtained in this study also indicates that 0.31 mg/cm^2^ silver can be recovered. This implies that the recovery of silver from X-ray and photographic films varied for different studies. This is probably due to the amount of silver remaining in the films during the imaging process.

## 4. Conclusions

Numerous studies have been conducted concerning the hydrolysis of gelatin and recovery of silver from X-ray films by using enzymes obtained from different sources including bacteria, fungi, and plants. However, there is no enzyme obtained from these sources that can remove gelatin from X-ray and photographic films except the enzyme used in this study, which is extracted from pineapple juice processing waste peel. The enzyme extracted from this source can remove gelatin completely and recover silver within 20–34 seconds. Until to date, no enzyme from any source can remove gelatin from X-ray film except the enzyme used in this study. This is an exceptional and novel enzyme used for gelatin hydrolysis reaction in different applications. The bromelain enzyme investigated in the present study shows unique properties unlike the bromelain enzymes studied before because this enzyme shows maximum activity at 70°C and pH 11.5, which is not observed before for other bromelain enzymes. Furthermore, the enzyme was characterized by extreme reusability (50th time) and efficiency of gelatin removal with a minimum concentration (0.54 U). The ability of the crude enzyme to retain its activity for repeated use makes it suitable for industrial applications. The use of 244U enzyme removed gelatin within 20 seconds, which is the first shortest time of gelatin removal recorded from X-ray films that are not observed in any chemical and enzymatic methods. Therefore, the crude bromelain enzyme extracted from waste pineapple peel was extremely efficient to remove gelatin from waste X-ray films. Since the enzyme recovered in this study worked in a vast range of pH and temperature values, it can be used for different applications. The cleared X-ray film can be reused for the manufacturing of X-ray film and used for the production of fabrics, packaging films and soft-drink bottles, and others. The results of this study showed that hydrolysis of gelatin and recovery of silver from X-ray films using bromelain are considered cheaper, pollution-free, and efficient. Thus far, crude bromelain extracted from waste peels not only recovered clean X-ray films for reuse, but it could also help to minimize the production cost of a large number of polyester films.

## Figures and Tables

**Table 1:** Gelatin removal efficiency of enzymes obtained from different organisms.

Enzyme source	Gelatin removal time	References
*Bacillus cereus* strain S8	15 minutes	[29]
*Bacillus subtilis* subsp. *subtilis*	30 minutes	[30]
*Bacillus subtilis* (NCIM 2724	4–6 days	[7]
*Purpureocillium lilacinum* LPS #876	6 minutes	[11]
*Aspergillus versicolor* PF/F/107	20 minutes	[12]
*Bacillus thuringiensis* sp.	1 hr	[31]
*Bacillus licheniformis* KBDL4	24 hr	[32]
*Vibrio* sp. R11	3 minutes	[10]
*Conidiobolus coronatus*	6 minutes	[1]
*Bacillus subtilis* ATCC 6633	15 minutes	[3]
Alkaliphilic *Bacillus* sp. B21-2	45 minutes	[33]
Alkaliphilic *Bacillus* sp. strain 18	2 minutes	[4]
Pineapple (*Ananas comosus*) waste (peel)	20 seconds	Present work

**Table 2:** pH stability of the enzyme at different preincubation periods.

pH value	Preincubation period											
	0 h		3 h		6 h		12 h		24 h		48 h	
	Gelatin removal time (seconds)	Residual activity (100%)	Gelatin removal time (seconds)	Residual activity (%)	Gelatin removal time (seconds)	Residual activity (%)	Gelatin removal time (seconds)	Residual activity (%)	Gelatin removal time (seconds)	Residual activity (%)	Gelatin removal time (seconds)	Residual activity (%)
**3**	370	100	410	90.24	492	75.20	630	58.73	827	44.74	1230	30.08
**4**	49	100	55	89.09	65	75.39	81	60.49	129	37.98	250	19.60
**5**	44	100	45	97.78	46	95.65	45	97.78	46	95.65	80	55.00
**6**	38	100	39	97.44	41	92.68	42	90.48	45	84.44	51	74.51
**7**	34	100	34	100	36	94.44	35	97.14	36	94.44	39	87.18
**8**	45	100	47	95.74	50	90.00	55	81.81	60	75.00	480	9.38
**9**	45	100	45	100	46	97.83	45	100	45	100	51	88.24
**10**	46	100	46	100	46	100	47	97.87	48	95.83	50	92
**11**	109	100	116	93.97	143	76.22	208	52.40	463	23.54	600	18.16

Residual activity = (activity of sample after incubation/activity of sample before incubation) × 100.

## Data Availability

The data used in the study are publicly available.
